# *Brucella abortus*: determination of survival times and evaluation of methods for detection in several matrices

**DOI:** 10.1186/s12879-018-3134-5

**Published:** 2018-06-05

**Authors:** Rene Kaden, Sevinc Ferrari, Tomas Jinnerot, Martina Lindberg, Tara Wahab, Moa Lavander

**Affiliations:** 1 National Veterinary Institute, Uppsala, Sweden; 2 Swedish Forum for Biopreparedness Diagnostics, Umeå, Uppsala and Solna, Sweden; 3 Swedish Joint Laboratory for Food Safety and Biopreparedness, Uppsala, Sweden; 4 Department of Medical Sciences, Uppsala University, Uppsala, Sweden; 5 National Food Agency, Uppsala, Sweden; 6 Public Health Agency of Sweden, Solna, Sweden

**Keywords:** *Brucella abortus*, Limit of detection, Diagnostics, Survival time

## Abstract

**Background:**

*Brucella abortus* is a highly pathogenic zoonotic agent, tempting for the development of a rapid diagnostic method to enable adequate treatment and prevent further spread. Enrichment of the bacteria is often used as a first step in diagnostics to increase the bacterial number above the detection limit of the real-time PCR. The enrichment of *Brucella spp*. takes at least 3 days, which might be avoidable if sensitive PCR methods can be used. Since many matrices contain PCR inhibitors, the limit of detection (LOD) must be determined for each separate matrix.

Another aim of this study was the determination of survival of *Brucella abortus* in the analyzed matrices.

**Methods:**

The LOD for the detection of *B. abortus* in 14 matrices, relevant for human medicine, veterinary medicine and food and feed safety, was determined to evaluate the need of a pre-enrichment step prior to real-time PCR.

The survival of *B. abortus* in the spiked matrices was tested by plate count in a 7-day interval for 132 days.

**Results:**

The limit of detection for *B. abortus* in most matrices was in the range of 10^3^–10^4^ CFU/g for cultivation and 10^4^–10^5^ CFU/g for direct real-time PCR.

The survival time of *B. abortus* was less than 21 days in apple purée and stomach content and 28 days in water while *B. abortus* remained viable at day 132 in milk, blood, spinach and minced meat.

**Conclusions:**

A direct PCR analysis without enrichment of bacteria saves at least 3 days. However, the limit of detection between direct PCR and plate count differs in a 10 fold range. We conclude that this lower sensitivity is acceptable in most cases especially if quick analysis are required.

## Background

Brucellosis is a bacterial disease that can affect many different animal species. The bacteria are 0.6 × 1 μm sized Gram-negative coccobacilli that may grow facultative intracellularly.

Currently, there are 12 species described, most of which are highly host specific. *Brucella abortus* occurs mainly in cattle while *Brucella melitensis* occurs in goat and *Brucella canis* in dogs. *Brucella* infections are usually transmitted in animals through semen, aborted embryos and discharge but also by inhalation and oral intake. Infected animals rarely show symptoms but during pregnancy, fetuses may be aborted, which can also be followed by long-lasting vaginal discharge [1].

Some *Brucella* species such as *B. abortus* have a high zoonotic potential and are a common source of human infection. Human brucellosis caused by *B. abortus* is called Bang’s disease and is characterized by prolonged and recurrent undulating fever that can last for many months or even years if not treated. The infection may persist in brain or bone tissue. The mortality rate is low, but effective treatment is challenging and there is no prophylaxis. The infectious dose is 10–100 bacteria for *B. abortus*. Depending on the temperature, *B. abortus* is able to survive up to 114 days in tap water [1]. The most common routes of infection for human brucellosis are inhalation, or via skin wounds and mucous membranes (e.g. in contact with infected animals, in slaughterhouses or in laboratory settings) and ingestion of contaminated food, primarily unpasteurized dairy products. The bacterium is fastidious, and may persist in the environment for prolonged periods of time. Hence, it is of interest to have robust methods of analysis for several kinds of matrices that may harbor the bacterium, e.g. dairy products, feed and clinical animal samples.

The focus of this study was the laboratory need for robust methods for analysis of samples relevant for human medicine, veterinary medicine and food and feed safety. The aims were to determine the survival time of *Brucella* in several matrices and to evaluate analytical sensitivity as limit of detection (LOD) for detection of *Brucella* in these matrices by selective plate cultivation and real-time PCR on direct extractions from the samples.

## Methods

### Bacterial strain and cultivation

*B. abortus* biovar 1 544^T^ (ATCC 23448^T^, NCTC 10093^T^) were streaked from glycerol stocks (− 80 °C) onto Farrell agar [2, 3] and incubated at 37 °C with 10% CO_2_ for 4 days. Based on plate count experiments, it was determined that OD_600_ = 1 corresponds to approximately 5 × 10^9^ colony forming units (CFU) per ml.

### Spiking of matrices

Tenfold dilution series with a CFU ranging from 2 × 10^1^ to 2 × 10^5^ were prepared in physiological NaCl (0.9%) for spiking. The actual bacterial concentration was determined by plate count on Farrell agar [2, 3], and used as correction factors throughout this study.

The matrices for the spiking experiments were:

1. Food: low pasteurized milk (3.8–4.5% fat), minced meat, wheat flour, spinach leaves, apple puree, tap water, and ground white peppercorns.
2. Feed: hay and samples of feed mill scrapings (FMS).
3. Clinical samples: bovine vaginal e-swab, defibrinated sheep blood, bovine placenta, bovine semen, and bovine stomach contents.

The samples were chosen based on a clinical, veterinary and biosecurity perspective. Bovine semen and bovine vaginal e-swab were remaining lab samples from standard care of the animal obtained by veterinary standard procedures from living individuals. Bovine placenta was taken after birth. Bovine stomach content was provided by the Lövsta slaughterhouse (Uppsala, Sweden). Defibrinated sheep blood was ordered from Thermo Fischer Scientific. No animal was euthanized for the purpose of this study.

All the matrices were free from artificial preservatives. Each sample was spiked with 1 to 10^5^ bacteria per ml. For each matrix a non-spiked sample was used as negative control. Spiking was performed directly in case of liquid matrices while 2 g of the solid matter was weighed up in 50 ml falcon tubes, mixed with 18 ml physiological NaCl and homogenized by vortexing with 10 glass beads with a diameter of 3 mm, prior to the addition of bacteria. A volume of 250 μl of the swab liquid and 0.4 g placenta were used for analysis, due to access to limited sample amounts of these sample types. Cultivation and DNA extraction was done directly after spiking the matrices.

Cultivation was carried out on selective Farrell agar plates with dilutions of 10^− 1^ and 10^− 2^ of the samples that were spiked with 1 to 10^5^ bacteria per 1 ml in addition to the undiluted approach. A volume of 100 μl of each matrix and each dilution was spread on Farrell agar. Due to sample consistency and size, placenta and swab samples were incubated from an inoculation streak. Plates were incubated at 37 °C, in 10% CO_2_ for 4–5 days to allow for colony formation of *B. abortus.*

### Molecular analysis

DNA was extracted from 200 μl samples using the EZ-1 DNA tissue kit and the EZ-1 extraction robot (Qiagen). The placental samples were first incubated with proteinase K and G2-buffer (QIAGEN) at 56 °C for 15 min due to the high porosity of the material. All other samples were processed without proteinase K treatment. Prior to extraction, 195 μl of each sample were mixed with 5 μl seal herpesvirus 1 virions (PhHV-1) to a final concentration of 10^6^ virions per ml, as an internal process control (IPC) [4].

The PCR target sequence for *Brucella* was the IS711 intergenic spacer gene fragment present in all *Brucella* species [5]. The genome of *B. abortus* has 7 copies of IS711 of which one is truncated [6]. As the other common target, the 16S rDNA [7] only exist in *B. abortus* in 3 copies [8] the IS711-tageted real time PCR is expected to be more sensitive than the corresponding 16S based method. For the IPC, a gB-polymerase gene fragment was used as the target.

The real-time PCR was performed as described previously [9]. Amplification was performed in the ABI 7500 fast thermo cycler (Applied Biosystems) using 95°C initial denaturation for 5 min and 45 cycles of 95°C denaturation for 15 s followed by 60°C amplification for 60 s.

At least 3 no template controls (NTC) and 3 positive controls (clean *B. abortus* DNA) were applied in addition to the IPC to evaluate the real-time PCR. The real-time PCR data analysis was done using the ABI 7500 software version 2.0.6 with a manually selected threshold of 0.12. The real-time PCR was validated at the National Veterinary Institute (SVA) and at the Public Health Agency of Sweden (FOHM) (data not shown).

Each spiking experiment was performed in triplicates on three different days for statistical reliability. Additionally, each sample was analyzed in duplicates in the real-time PCR. Furthermore, the experiments were performed in 10-fold dilutions and thus the results of real-time PCR and cultivation should represent the concentrations of the dilution row in a logical 10-fold sequence.

The determination of the LOD with real-time PCR and culturing was performed according to the recommendations of the Minimum Information for Publication of Quantitative Real-Time PCR Experiments (MIQE) guidelines [10] and the US food and drug administration (FDA) [11], respectively. This means for the evaluation of the results of this study, that the LOD was determined by the result in which 95% of the real-time PCR samples gave a positive quantification cycle (cq) signal and in which a number of 25 to 250 CFU was counted on the agar plates. Furthermore, the recovery rate was determined in % where 100% is equal to the resulting concentration of bacteria spiked to the samples. The lowest limit of a possible single detection (LLD) was calculated according eq. 1 for cultivation experiments and determined as single occurrence of one positive cq value in qPCR independent on reproducibility and confidence level. The LLD is also applicable to estimate the minimal required sample weight for a positive detection.

$$ \mathrm{LLD}=\frac{{\mathrm{load}}_{\mathrm{min}}}{\mathrm{n}\ \mathrm{grown}\kern0.17em \mathrm{bacteria}}\kern0.5em {\mathrm{Load}}_{\min \dots}\mathrm{spiked}\ \mathrm{load}\ \mathrm{of}\ \mathrm{bacteria}\ \mathrm{on}\ \mathrm{the}\ \mathrm{plate}\ \mathrm{with}\ \mathrm{the}\ \mathrm{lowest}\ \mathrm{number}\ \mathrm{of}\ \mathrm{detected}\ \mathrm{bacteria};{\mathrm{load}}_{\mathrm{min}}=\mathrm{LOD}\ {\mathrm{if}\ \mathrm{load}}_{\mathrm{min}}>25 $$

Equation 1: Calculation of the lowest possible limit of detection

### Survival analyses

Samples with a final concentration of 10^4^ bacteria per g of matrix as described above were stored at 4°C. A weekly sampling with selective cultivation was performed for 132 days. The samples were spread undiluted and in a dilution of 1:10^− 1^ and 1:10^− 2^, incubated on selective Farrell agar as described above, and the number of CFU was determined according FDA’s recommendations [11].

## Results

For the evaluation of a method for the detection of *B. abortus* and determination of the survival of the bacterium in food, feed and clinical samples, several matrices were spiked with various concentrations of bacteria. The recovery rate was determined by plate count and real-time PCR.

*Brucella abortus* is a representative species of the genus Brucella due to the high occurrence and the impact that this organism causes worldwide. Thus, *B. abortus* biovar 1 544^T^ (ATCC 23448^T^, NCTC 10093^T^) was used for the experiments of this study.

**Fig. 1:**
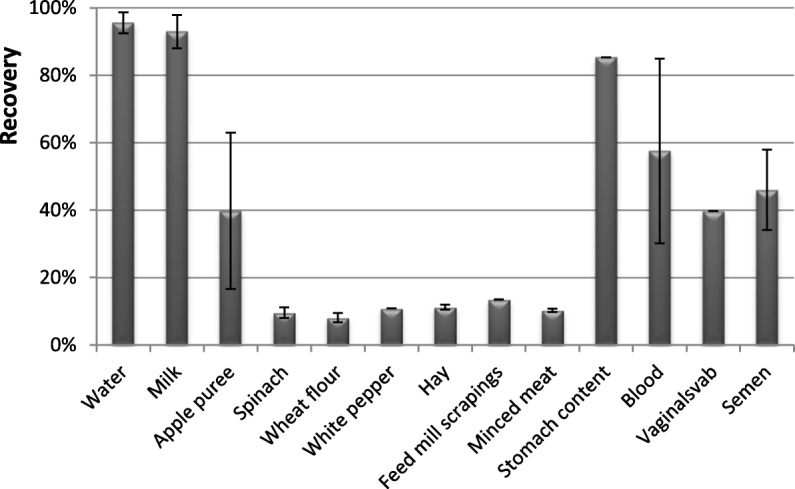
Recovery obtained by cultivation. Legend: Recovery of *Brucella abortus* from spiked samples; 100% is equal to the initial concentration of bacteria spiked to the samples

**Fig. 2:**
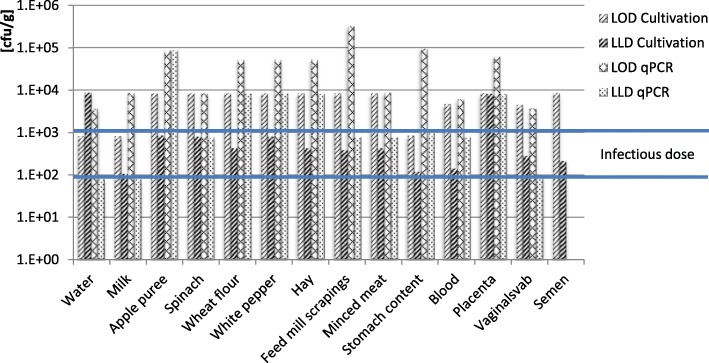
LOD and LLD [cfu/g]. Legend: Limit of detection (LOD) and lowest possible limit of detection (LLD) of *Brucella abortus* in several matrices obtained by cultivation and real-time PCR

*B. abortus* could be cultured from all different matrices. The recovery by cultivation one hour after spiking was highest in water and milk with more than 96 and 93% recovery respectively (Fig. 1). Milk is a natural reservoir of many *Brucella* species [12] and *B. abortus* persisted throughout the study for 132 days with a number of colony forming units (CFU) of 4 × 10^4^ per ml, which is almost the same CFU as at the beginning of the experiment. While the statistical valid LOD obtained with cultivation in milk (825 CFU/ml) was lower than the LOD obtained using real-time PCR on extractions made directly from the samples (8667 CFU/ml) (Fig. 2), the LLD of both methods was approximately 100 CFU/ml.

**Fig. 3:**
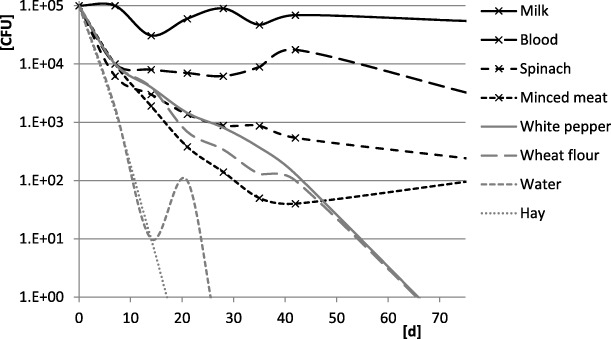
Survival of *Brucella abortus.* Legend: Survival of *B. abortus* in different matrices during the first 70 days of the study. *B. abortus* remained viable during days 71–132 in milk, blood, spinach and minced meat

In tap water, which is the least complex of the studied matrices, the recovery rate was almost 100% (Fig. 1) and the LOD was comparable with the LOD in milk (Fig. 2). The survival time of *B. abortus* in tap water was 28 days. A rapid decrease of the CFU in water was observed in the beginning of the experiment (Fig. 3).

The recovery rate in stomach content from the abomasum with a naturally low pH was 85% but only 50% in the clinical samples: blood, semen, and vaginal swab. An even lower recovery rate with 10% was observed in spinach, hay, FMS, white pepper, wheat flour, and minced meat (Fig. 1).

## Discussion

While the general expected survival time of *B. abortus* in milk according to former reports is no longer than 87 days [13, 14] the bacteria survived 132 days in our study. The differences could be caused by different types of milk or different storage conditions within the experiments.

The LLD obtained from cultured and uncultured milk samples was approximately 100 CFU/ml, which is in agreement with the results published by MER Hamdy and AS Amin [15]. However, a higher fat content of milk should correspond to a higher content of casein micells, which are binding DNA. This phenomenon might explain the factor 10 between the culture-based and PCR-based LOD in milk samples.

*B. abortus* survived in tap water for 28 days in our study. Falenski et al. determined a survival time of *B. abortus* in mineral water of 63 days [13]. The tap water used in our studies was treated with sodium hypochloride, which probably caused the shorter survival time.

The recovery rate from stomach content was 85%. Most *Brucella* infections occur orally and the most relevant source of human infections is unpasteurized milk. Thus, *B. abortus* has probably evolutionally adapted to the low pH in the gastro-intestinal tract. Due to the high recovery rate of the bacterium from stomach content, this source of infection should be recognized in healthcare, even if the risk is limited to individuals with acute intake of *Brucella spp.* as it may occur in *Brucella*-endemic countries.

The clinical samples: blood, semen, and vaginal swab are known to contain substances that affect the recovery rate of bacteria. This also applies to placenta, which, in addition, has a surface texture that increases the total surface. Thus, the LOD for placental samples was 8250 CFU/g which is high but, in terms of actually occurring CFU in infected individuals, sufficient for diagnostics.

Hay and FMS may contain naturally occurring nanoparticles that commonly have charged edges or surfaces [16]. Those particles are able to bind or affect the negatively charged bacteria and nucleic acids [17]. The high LOD in hay and FMS obtained by real-time PCR analysis supports this theory. The presence of bi- or multivalent cations or humic substances enhances the described effect due to the increased number of possible binding sites.

The highest standard deviation within the culture experiments was observed in blood and apple puree. The same batch of both matrices was used for all independent experiments. Thus, the observed differences might be caused by inhomogeneity of the matrices and therefore corresponds with the expected variance of results in real samples. The composition of blood varies day by day even within the same individual which leads to the conclusion that it is challenging to create standardized blood samples. Without this standardization and with the presented standard deviation of our experiments it should be discussed if blood containing media or blood culture, which is a common method not only in *Brucella* diagnostics, could be replaced by more stable standardized methods in the future.

Some spices and herbs of the present study are known to have an antimicrobial activity. Spinach contains the antimicrobial peptides So-D1–7 that might contribute to the low recovery rate [18]. The inhibitory effects of white pepper on bacterial growth observed in this study was also described by E Ceylan and DY Fung [19]. Wheat is known to contain inhibitory peptides, thionins [20]. However, the specific effect of these inhibitory substances was not tested against *Brucella*. The results of this study indicate an inhibitory effect of these matrices on growth and survival of *B. abortus* but further investigations are necessary to confirm this observation.

## Conclusions

The evaluated method is time saving due to the direct application of the real-time PCR without an enrichment of bacteria while detection of *B. abortus* by cultivation was in some cases more sensitive. The limit of detection was in a range of 10^3^–10^4^ CFU/g for cultivation and 10^4^–10^5^ CFU/g for direct real-time PCR with exception of the higher LOD of FMS. The lowest possible limit of detection LLD of with real-time PCR was the most sensitive method and the only method to detect *B. abortus* in concentrations near the infection dose. Since LLD is based on a single positive signal the method is appropriate for a first screening. As a result of this study we recommend using real-time PCR-LLD directly. In case of a negative signal, we recommend the treatment of samples and cultivation of bacteria as described in the methods section.

Furthermore, we present the survival times of *B. abortus* in several matrices, which was less than 21 days in apple puree and stomach content and 28 days in water, while *B. abortus* survived for more than 132 days in milk, blood, spinach and minced meat.

## Abbreviations

ATCC: American Type Culture Collection
CFU: Colony forming units
FDA: US food and drug administration
FMS: Feed mill scrapings
FOHM: Public Health Agency of Sweden
IS711: Intergenic spacer 711
LLD: Lowest possible limit of detection
LOD: Limit of detection
MIQE: Minimum Information for Publication of qPCR Experiments
MSB: Swedish Civil Contingencies Agency
NaCl: Sodium chloride
NCTC: National Collection of Type Cultures England
NTC: No template control
OD_600_: Optical density at wavelength 600 nm
PhHV-1: Phocine (seal) herpesvirus 1
qPCR: Quantitative polymerase chain reaction
SVA: Swedish National Veterinary Institute
